# Biomarkers and mechanisms associated with cancer‐induced cardiac cachexia: A systematic review

**DOI:** 10.1002/jcsm.13267

**Published:** 2023-05-21

**Authors:** Lisa Bagnall, Oliver Grundmann, Marilyn G. Teolis, Saun‐Joo L. Yoon

**Affiliations:** ^1^ James A. Haley Veterans' Hospital & Clinics Tampa Florida USA; ^2^ Department of Medicinal Chemistry, College of Pharmacy University of Florida Gainesville Florida USA; ^3^ Department of Biobehavioral Nursing Science, College of Nursing University of Florida Gainesville Florida USA

## Introduction

Cancer cachexia is a severe multifactorial syndrome^1^ that affects up to 80% of patients with advanced cancer, causing death in 20–80% with no effective treatments.^2^, ^3^ It causes multi‐organ alteration and loss of skeletal and cardiac (myocardium) muscle mass.^4^, ^5^ Cardiac muscle wasting may result from cardiac protein loss associated with increased oxygen consumption and energy expenditure, resulting in cardiac insufficiency.^5^ It is hypothesized that significant tissue inflammation and oxidative stress during cancer progression cause cardiac wasting‐associated cardiomyopathy, such as a thinned ventricular wall, local tissue hypoxia and arrhythmias.^6^ This review provided available evidence of cancer‐induced cardiac cachexia in human and non‐human models by examining biomarkers and the contributing factors to the development and progression of cardiac cachexia. Investigation of the potential biomarkers affecting cardiac muscle wasting is essential for improving patient outcomes.

## Methods

### Search strategy and selection criteria

**Figure 1:**
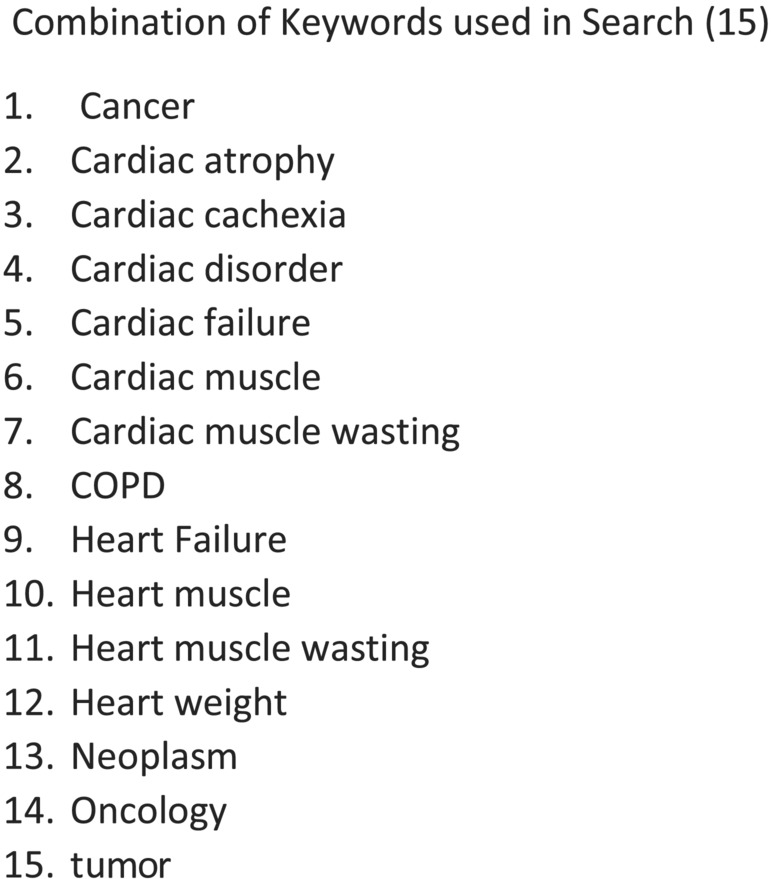
Combination of terms or keywords used to retrieve the articles for this review.

**Figure 2:**
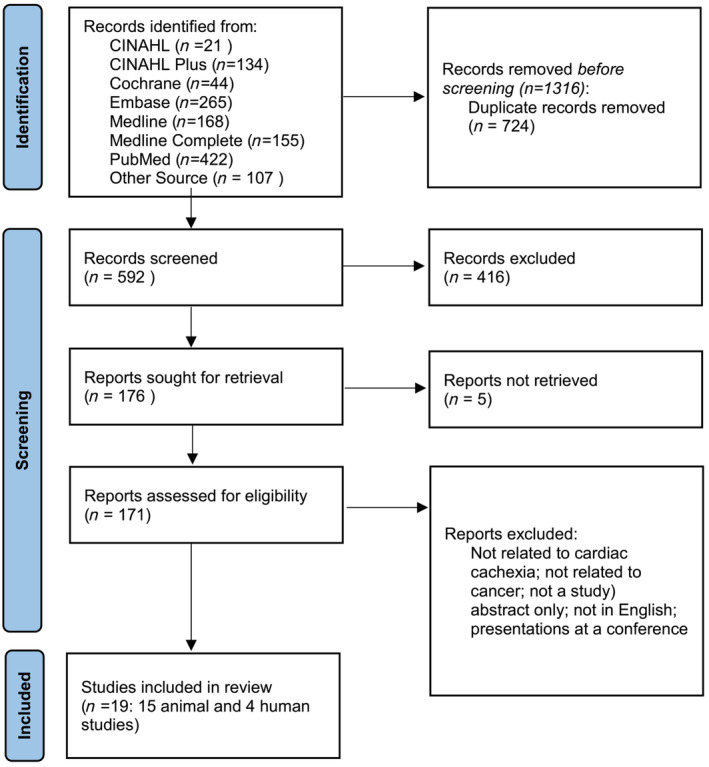
PRISMA flow chart.^7^ The PRISMA flow chart was obtained from the PICO Portal program and shows the number of studies identified in each database, how many met criteria and the number that was included in the review. The number of records excluded is also shown. The reason for excluding reports/studies is described on the lower right.

The search strategies included text words and medical subject headings related to cardiac cachexia in human and animal oncology studies (*Figure* *1*) in PubMed, Embase and the Cochrane Library. The databases in the EBSCO Discovery Service (EDS) were Ovid MEDLINE; MEDLINE and CINAHL Complete; and CINAHL Plus with Full Text. The years of publication were from 2011 through March 2021 limited to English‐language publications. The authors included randomized controlled trials and retrospective, prospective, descriptive, cadaver and animal studies. There were 15 animal and 4 human studies in this review that met the eligibility criteria (*Figure* *2*).

## Results

### Animal studies

**Figure 3:**
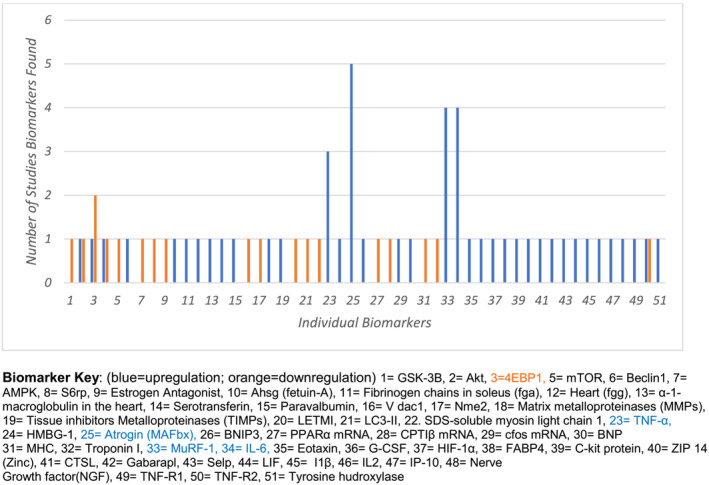
Frequencies of upregulated and downregulated biomarkers in animal studies. This figure shows the frequencies of upregulated (blue) and downregulated (orange) biomarkers involved in cancer‐induced cardiac cachexia. The biomarker key identifies the individual biomarkers. Atrogin, IL‐6, TNF‐α and MuRF1 were the most frequently upregulated biomarkers.

**Figure 4:**
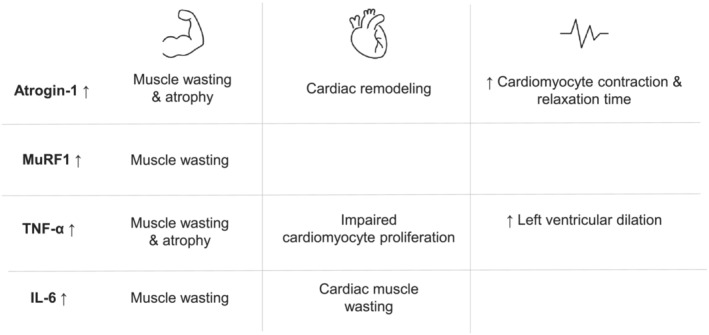
This figure highlights the effects from the individual upregulated biomarkers seen in cancer‐induced cardiac cachexia.

We reviewed the most discussed biomarkers and their change in gene expression (blue‐coloured bars in *Figure* *3*) affecting cardiac cachexia pathways in animal studies. These were atrogin‐1 (muscle atrophy F‐box), muscle RING‐finger protein‐1 (MuRF1), tumour necrosis factor‐alpha (TNF‐α) and interleukin‐6 (IL‐6). *Figure* *4* shows the impact of these upregulated biomarkers on cardiac tissue and conductivity.

#### Genes regulating cardiac muscles

Among many genes that regulate skeletal and cardiac muscles, some only affect cardiac muscle, whereas others regulate skeletal muscle growth, maintenance and atrophy. More genes affect skeletal muscle than the myocardium, and very few of the same genes regulate both muscle types.^8^ Mice injected with colon cancer cells lost approximately 20% of weight post‐inoculation within 14 days. A total of 110 genes were upregulated during cardiac, whereas 815 genes were upregulated in skeletal muscle wasting.^8^ Of these genes, only 76 common genes upregulated and 18 genes downregulated cardiac and skeletal muscles.^8^ Sixty‐four genes downregulated the cardiomyocytes, whereas 624 genes downregulated the skeletal muscles. Interestingly, some genes upregulated the cachectic heart but downregulated skeletal muscles, and vice versa.^8^ Gene regulation demonstrated the complexity and marked differences in skeletal and cardiac muscle regulation.^8^

#### Atrogin‐1

Atrogin‐1 acted to tag proteins for proteolysis by attaching ubiquitin, leading to muscle atrophy in cancer. It facilitated cardiac remodelling and muscle wasting, hallmarks of cardiac cachexia.^4^ Atrogin‐1 gene expression was upregulated in 5 out of the 15 animal studies.^7^, ^9^, ^10^, ^11^, ^12^

A study of 24 female CD2F1 mice inoculated with colon C26 adenocarcinoma cells examined in vivo heart function, in vitro cardiomyocyte and biomarkers of muscle proteins.^9^ In vitro cardiomyocytes were increased in tumour‐bearing mice, and the time for sarcomere contraction and relengthening to 90% was increased compared with the control group.^9^ Atrogin‐1 and Bnip3 gene expressions were significantly increased in the heart muscle of tumour‐bearing mice. Cardiomyocyte peak contraction and relaxation times were increased.^9^ Systolic failure was associated with an increased relaxation time.^9^ The posterior wall thickness of the heart was substantially reduced in the tumour‐bearing mice compared with control mice. No differences were found in overall cardiac function, heart rate or left ventricular diastolic diameter between the tumour and control groups.^9^

In male CD2F1 mice with C26 adenocarcinoma cells inoculated, atrogin‐1 was upregulated in the heart along with proto‐oncogene c‐fos mRNA, brain natriuretic peptide (BNP) and IL‐6. Peroxisome proliferator‐activated receptor alpha (PPARα), collapse section carnitine (CPTIβ) mRNA, myosin heavy chain (MHC) and troponin gene expressions were downregulated.^10^ Mammalian hearts rely on lipid oxidation for energy. However, when the amount of energy to the heart is decreased due to the downregulation of PPARα and CPTIβ, glucose becomes the primary cardiac muscle energy source. This was suggested as a reason for reduced functioning, as glucose cannot serve as a long‐term energy source.^10^ The hearts of adenocarcinoma‐bearing mice showed significantly higher mRNA expression of MuRF1 and a higher gene expression of atrogin‐1. This was consistent with a high level of protein ubiquitination, resulting in cardiac protein loss and cardiac muscle wasting.

#### Muscle RING‐finger protein‐1

MuRF1 gene expression was upregulated in five studies.^7^, ^10^, ^11^, ^12^, ^13^ In one study, mice were injected with pancreatic cancer cells into the subcutaneous space, the peritoneum or the tail.^13^ The severity and progression of tumour development depended on the injection site with peritoneal and tail injections, leading to more severe cachexia than subcutaneous administration.^7^ Male mice in this study developed larger tumours than female mice. Both sexes developed cardiac atrophy with increased expression of cardiac muscle genes Bnip3, Cathepsin L (Ctsl) and Gabarapl. Greater cardiac muscle loss was observed in male adenocarcinoma‐bearing mice, indicating that estrogen may have a protective cardiac effect in females.^13^ There was reduced gene expression of MAFbx and protein expression of MuRF1 in the myocardium compared with skeletal muscle.^7^ MuRF1 protein (4.3‐fold) and Atrogin (same as MAFbx gene) (3.8 fold) expressions were significantly higher in the hearts of mice with cancer cachexia than controls, indicating upregulation of the ubiquitin proteosome system (UPS).^12^ The increased oxidative stress is linked to higher expression of xanthine oxidase and NADPH oxidase, releasing reactive oxygen species (ROS), leading to loss of functioning myocardium. Its mechanism is distinctly different from skeletal muscle loss, leading to cardiac atrophy.^12^

#### Tumour necrosis factor‐alpha

Tumour necrosis factor‐alpha (TNF‐α) was upregulated to accelerate cardiac cachexia in four animal studies.^11^, ^14^, ^15^, ^16^ Cachectic mice with CT‐26 colon cancer cells had a significant reduction by 62% in heart weight coupled with 1.9 times larger left ventricular dilation and a 72% reduction of cardiomyocyte area than control mice, which indicated cardiac atrophy in this model.^14^ Rats injected with ascites fluid from CT‐26 cachectic mice had an impaired cardiomyocyte proliferation and a 62% reduction of cardiomyocyte maturity associated with oxidative stress through increased amounts of 8‐hydroxydeoxyguanosine and mitochondrial damage.^14^ Accompanying these alterations in cardiac remodelling, TNF‐α was upregulated 2.3‐fold in the cachexia model compared with the control group, indicating the critical role of pro‐inflammatory cytokines in cachectic signalling pathways and increased energy metabolism.^14^

TNF‐α was also upregulated in a mouse model of Lewis lung carcinoma, along with IL‐6, nerve growth factor mRNA, TNF receptors 1 and 2 and the rate‐limiting enzyme for catecholamine synthesis, tyrosine hydroxylase.^16^ There were no differences in mitochondrial or nuclear volumes nor the heart function between the lung cancer and control groups based on echocardiography.^16^

#### Interleukin‐6

Interleukin‐6 (IL‐6) was upregulated for cardiac cachexia in four animal studies.^10^, ^11^, ^15^, ^16^ Mice injected with colon cancer cells (C26) plus leukaemia inhibitory factor (LIF) showed upregulation of TNF‐α and the same reduction in heart mass as mice not receiving LIF.^15^

TNF‐α and IL‐6 were upregulated in mice with C26, regardless of C26 injection sites (i.e., peritoneum or subcutaneous space), which indicated an inflammatory response related to increased reactive oxygen species (ROS) generation.^11^ In response to ROS, UPS‐related upregulation of atrogin‐1 and MuRF1 increased in the myocardium of mice with C26 injection into the peritoneum. Only mice injected with C26 into the peritoneum showed heart muscle mass wasting.^11^ This suggested that the environment where tumour cells grew may affect how cachexia developed in various muscle groups.^11^

#### Sarcomeric and metabolic proteins

Sarcomeric proteins, an anchor for myofibrilla proteins and the structure holding sarcomeres together, are mostly upregulated, and metabolic proteins are downregulated across muscle types in cachectic conditions.^17^ The M‐lines and Z‐discs within the sarcomeres of C26‐bearing mice disintegrated.^17^ In cachectic heart muscles, mitochondria were swollen and had electron‐lucent areas. This led to the loss of cristae and resulted in the depletion of adenosine triphosphate.^8^ The energy proteins in the heart were upregulated and included Ag1, Pygm, Tpi1 and Uqcrq.^8^ These responses may have served to preserve heart function.^8^ Metabolic proteins downregulated in cachectic mice included voltage‐dependent anion‐selective channel 1 (Vdac1) and Nme2, a gene that suppressed apoptosis. The reduced energy and density of the mitochondrial matrix led to cardiac cachexia or muscle wasting.^17^ Morphological alterations in the mitochondria, disruption of sarcomeric structure in the heart and upregulation of inflammation‐related proteins such as Ahsg (fetuin‐A), Fga and Fgg drove muscle catabolism.^17^

#### Matrix metalloproteinases

Matrix metalloproteinases (MMPs) break down proteins and play a role in wound healing, angiogenesis and tumour cell metastasis. MMP gene expressions were upregulated in C26 tumour‐bearing mice, and collagenase MMP‐9 was upregulated at the protein level.^18^ MMPs remodelling of the extracellular matrix resulted in a significant increase in the amount of collagen deposits in the heart muscle.^18^ The collagen by‐product, hydroxyproline, was increased in the serum of C26 tumour‐bearing mice, suggesting increased collagen turnover.^18^ Remodelling and decreased wall thickness from the upregulation of MMPs may result in cardiac dysfunction and heart failure in C26 tumour‐bearing mice with cachexia.

### Human studies

Human studies have not been able to evaluate specific cardiac biomarkers as seen in animal studies because it would not be ethically reasonable to obtain a cardiac biopsy for this purpose. All biomarkers mentioned in the animal research section have never been verified in human subjects. Most human studies were retrospective and observational and mostly analysed cardiac function and patient symptoms.

#### Cardiac symptoms associated with cachexia status

Cardiovascular symptoms indicated cachexia status in one human study.^19^ A total of 103 subjects (male:female = 11:92) with newly diagnosed stages of II–IV malignancies were classified as non‐cachectic, pre‐cachectic or cachectic in this study.^19^ Non‐cachectic cancer patients showed the least number of cardiovascular symptoms. Those with pre‐cachexia and cachexia had the most cardiac findings such as high‐grade premature ventricular contractions (PVCs), hypertension and either new or progressive chest pain secondary to coronary artery disease.^19^

#### Melatonin

Endogenous melatonin excretion was greatly reduced in patients with cancer and cancer‐associated cachexia, who developed cardiac dysfunction.^19^ The decrease in melatonin production, measured by urine 6‐sulfatoxymelatonin (aMT6s),^19^ became more pronounced (*P* < 0.05) in pre‐cachectic and cachectic subjects. This effect was even greater in individuals 60 years and older.

#### Cardiac dysfunction associated with advanced cancer

When comparing patients with advanced pancreatic or colorectal cancer (*n* = 43) to a healthy control group (*n* = 18) and a heart failure group (*n* = 37),^20^ patients with cancer had increased blood pressure, cardiac output (CO) and stroke volume (SV) with a higher maximal rise in left ventricular pressure (Dp/dt_max_) than the control group and had significantly lower body weight (*P* = 0.002) than those in the heart failure group (*n* = 37).^20^ SV decreased with age in the heart failure and cancer groups.^20^ CO correlated with age in the heart failure and cancer groups and with body weight in the control group.^20^

#### Heart weight comparisons in post‐mortem studies

Post‐mortem analysis comparing heart function and weight in cachectic patients with gastrointestinal (GI), lung and pancreatic cancer to non‐cachectic patients indicated a significantly decreased heart weight,^9^ suggesting cardiac wasting through inter‐tissue and inter‐organ crosstalks.^4^, ^5^, ^21^

Cardiac wasting by measuring the heart weight and left and right ventricular wall thickness was examined in 177 patients who died of cancer (58 with lung cancer, 60 with pancreatic cancer and 59 with GI cancer other than pancreatic cancer).^22^ Of those, 54 (30.5%) showed cancer‐associated cachexia. Patients with cancer‐associated cachexia had a significantly lower heart weight than non‐cachectic patients (*P* < 0.001) and patients with non‐cancer and non‐cardiovascular diagnoses (*P* < 0.05). Heart weight and lower left and right ventricular weight were not significantly different among cancer types regardless of cachexia status, indicating that cardiac cachexia is a general concern independent of cancer type.^22^

## Discussion

Our knowledge about the causes of cardiac cachexia in cancer remains limited and the possible mechanisms have only been explored in animal studies to date. A better understanding of the pathophysiology of cardiac cachexia may help to determine if targeted therapies can effectively block the upregulation of various genes and cytokines that initiate and facilitate cancer‐induced cardiac cachexia. Understanding the distinct nature of cancer‐related cardiac cachexia and what distinguishes it from other causes may also lead to finding targeted, effective treatments. Treating cardiac cachexia early and before clinical changes are noted may improve overall cardiac function and lead to better patient outcomes.

## Conflict of interest statement

The authors have no conflicts of interest.
